# The Painless Separation of Teeth

**Published:** 1913-04-15

**Authors:** R. Ottolengui


### THE PAINLESS SEPARATION OF TEETH.

BY R. OTTOLENGUI, D.D.S.

In the January issue of *Items of Interest* Dr. Van Woert described a method of painlessly separating teeth. As it is our belief that a correct comprehesion of technical details of operations which involve mechanical processes, can best be conveyed with illustrations which appeal to the eye thus aiding the verbal description, we used no less than four illustrations in order to make clear the several steps of the method. We believed, and still believe, that we presented to our readers, and to their patients, a valuable New Year's present in thus publishing and illustrating the very last word in the matter of separating teeth painlessly and expeditiously. Within a week Dr. Van Woert had received about a hundred letters from dentists and from dental dealers asking where the silk might be obtained. This shows that the majority, at least, understood what Dr. Woert was endeavoring to teach.*

*The method described by Dr. Van Woert is really Ottolengui's suggestion. It consists of drawing a loop of French silk twist through the approximal space by means of a fine ligature acting as a leader, from the buccal to the lingual side of the teeth. One of the free ends of the silk is then carried over the crown surfaces and passes through the loop that has been drawn through between the teeth to the lingual surface. This forms a knot which when drawn up tight wedges the silk twist between the approximating teeth at the contact points, where it is securely tied and the surplus ends cut off and the patient dismissed. The silk is such as is used by the orthodontists in regulating teeth and may be secured from the dental supply houses —Editor of *Dental Register*.

But alas for human nature! Many of us are so enamored of our own methods, that we scarcely give sufficient attention to the other man, telling of his way, to really grasp his idea. For example, one gentleman, in perfect good faith, and assuredly with the kindest motives of helpfulness writes as follows to Dr. Van Woert:

### SEPARATING WITH TAPE.

He declares that he uses "ordinary sewing tape," and adds: "It has all the advantages of the ligature silk of which you speak (sic?) and which I used previously to using the tape, and to my mind has the following additional advantages. The entire operation can be done by the patient, thereby taking none of the dentist's time, and any amount of separation can be gained, etc."—"The method is as follows: If the patient is to be seen on Wednesday, I give him a piece of tape and tell him to put it between the teeth on Monday night and cut it close to the tooth, both front and back. Tuesday morning, put in two thicknesses; Tuesday night three thicknesses, and Wednesday morning four. Unless the teeth are very close together the patient can do this very easily. If too tight together for him to do it I use the immediate separator and put the first thickness in myself," etc.

An analysis of the above shows that the writer either scarcely read what Dr. Van Woert wrote, or else did not understand either the text or the illustrations. First he mentions that he had used the ligature silk "of which you speak" and had abandoned it for the tape. The tape, used as described by this gentleman is certainly effective and when first introduced to the profession, probably a quarter of a century ago, was an advance over previous methods, namely rubber, or orange-wood. But no one who had ever used the silk "of which Dr. Van Woert speaks," using it in the manner described in his paper, would ever have abandoned it for tape. It is probable that the writer never having seen the silk of which Dr. Van Woert writes, has confounded it with silk used by dentists for ligaturing the rubber dam about the necks of teeth, whereas Dr. Van Woert was alluding to a French silk, now used by orthodontists in ligating teeth to orthodontic appliances. The common dental floss silk *stretches* when moistened, whereas this French silk *shrinks* when wet. Of this more presently.

Then the writer describes his way of using the tape. He gives it to the patient with instructions for removing and replacing three times. The method presented by Dr. Van Woert is such that the ligatures could be tied between the teeth in less time than would be required for explaining the tape method to the average patient. Moreover, the patient would not be burdened with any part of the work, but could go home and forget all about the separation. Again, the separation will be between the proper teeth, which is not always true when put in by the patient. But finally, the writer admits that when the teeth are very tight together he uses an immediate separator and inserts inserts the first tape himself. It is just these teeth that are very tight together that most need separating and the ligature could be applied painlessly in half the time needed to adjust the immediate separator, *which always causes pain*. If any dentist is inclined to dispute this, before doing so, let him have his assistant use the immediate separator in his own mouth.

A short time after the publication of Dr. Van Woert's paper Dr. Van Woert dined with a number of friends, all very prominent men. He was greeted with compliments on his paper, and then one after the other the men made remarks of this character: "That is a fine way to separate teeth; I have been doing it for twenty years!" "Say, Van Woert, that *is* a painless way to separate teeth, but what makes you think it is new?" "I am glad, Van Woert, that you have illustrated that use of the ligature for separating teeth; I have thought of describing that myself for years." "Oh! Van Woert, I am glad to see that you have at last discovered that you can separate teeth with silk. Sorry for your patients, if you have been hammering orangewood between their teeth all these years!"

And so they all had a bit of fun with him for a while, but when they had taken their seats, Dr. Van Woert arose and said: "Now, my good friends, I'll wager a dinner for this party that not one of you have ever used that method of separating teeth; that you have never seen the silk; and that if I gave you a piece you would not know how to tie the knot, without again studying the illustrations."

The challenge rather sobered the company, and Dr. Van Woert then demonstrated the tying of the knot, using a bit of cord, and tying it between his fingers, whereupon all admitted that they never had used that particular silk in that particular way.

And that is "the milk in the cocoanut." The method depends for success upon the use of this particular silk, tied in this particular manner.

And this story demonstrates that even men of national reputation and of undoubted skill may read inattentively, and thus lose the point of the story.

Another correspondent wrote that the method is good, but old. He said that it had been given to the profession by Dr. J. Austin Dunn of Chicago, and could be found described in the *Dental Review* for 1892. This certainly was interesting because it was so precise. The reference was consulted, and it is an article half a page long, with two illustrations, so there can be no mistake about what is meant. It is the well-known method of placing a bit of absorbent cotton between the teeth, and tying the same with a silk ligature, which is passed under the cotton and then tied above the contact point. This also is a good method in many cases, but observe that it is the *cotton* that separates the teeth, the silk being used only to hold the cotton in place. In the new method, *for it is new*, it is the contraction of the ligature itself which spreads the teeth apart, and this is greatly aided by the method of tying.

To those who may think that the methods are the same, the following experiment is suggested. Take a sensitive miss of fourteen, with the second permanent molars *recently erupted.* Then endeavor to tie cotton, in the manner described in the *Dental Review,* first, between the second bicuspid and the first molar, and then between the two molars. The experimenter will discover with what pleasure the patient will endure his efforts, first to place the ligature, and then to pack enough cotton between the tightly placed teeth so that it may subsequently be tied in place. For it is evident that where teeth are in very close contact, not very much cotton can be packed through the contact point; it must be poked under it, painfully disturbing the sensitive gingiva. On the other hand, it is but fair to state that in the presence of large cavities, this cotton method is most desirable, but it will be more efficacious used in conjunction with the method described by Dr. Van Woert than with ordinary dental floss.

### HISTORY AND RATIONALE OF SEPARATING TEETH.

It may be profitable to consider the various methods for separating teeth, which have been evolved. First we should take cognizance of the anatomy of the parts. We have to deal with two bony structures, set in bone, and surrounded by pericementum. Between these we find the gingiva filling the interproximal space. This space is triangular in form, the base of the triangle being toward the soft tissue, and the apex at the contact of the two teeth. Pain is of two characters. It may result from exerting sudden and great stress against the pericementum, pinching it by forcing the tooth root against the socket wall. It may likewise be caused by stress against the soft tissues.

Probably the first attempt at making space was by driving a piece of wood between the teeth, the teeth moving apart through absorption of moisture and consequent swelling of the wood.

Then we had the use of pure rubber wedges. (The exact sequence of these two methods is not important.) The rubber wedge spread the teeth by exerting elastic force. These two methods had a fault in common. When first inserted, the wooden or the rubber wedge is held in place between the contact points, but as the teeth yield there is a tendency, greater in the rubber than in the wood, for the wedge to slide toward the base of the triangle, thus impinging against the soft tissue, causing pain, and often serious injury.

Next (probably) we had the absorbent cotton wedge, first used in the form of a twist, or rope, inserted dry, and later packed between the teeth after first impregnating it lightly with sandarach varnish. Following this, of course, came the more efficient method of tying the cotton to place with silk ligature. This not only kept the cotton in position, but was supposed to force it toward the contact point and keep it away from the soft tissues. It is this feature which becomes more efficacious when the new silk, which *shrinks*, is used instead of dental floss which *stretches*.

Coincidentally, or immediately after the cotton wedge, came the wedge with tape. All improvements in methods are by evolution, and it is a common rule with historians to determine the priority of invention or construction by deciding which is the more primitive, the improved product usually coming last. Thus it is likely that the cotton wedge antedated the tape, the latter being adopted because it could more easily be forced between two teeth, while being made of cotton would have the same force in spreading by swelling upon absorption of moisture.

Then, just as the cotton wedge was steeped in sandarach to keep it from becoming foul, someone in the West dipped cotton tape in wax, and waxed tape is still much used in Chicago and vicinity. In New England, Dr. Frank Blivin of Worcester, presented a tape steeped in gutta percha, which is better than any other tape method.

The various steel appliances for immediate separation are familiar to all, but can scarcely be considered in a discussion of painless separation of the teeth.

ORTHODONTIC METHODS OF SEPARATION.

It will be observed that all the methods thus far mentioned have been used mainly for separating teeth which need filling. But the orthodontist often found that it would be easier to fit molar bands if he might first obtain a little space. He also quite quickly discovered that none of the existing methods would serve to open the space mesially of the first molar, and more especially the space distal to that tooth. Men who at that period were using the Angle method of ligating the teeth to the expansion arches with wire ligatures, would pass a wire ligature under the contact points, bring the end out buccally and twist the two ends tight, thus contracting the ligature around the contact points. This was effective, even though not always painless. For in this connection there is an axiom that should never be forgotten.

*When the greatest stress is made to bear against the greatest resistance, the maximum of pain is caused.*

Measured by this rule, the method is faulty, because the ligature is tied as tight as possible at the moment of its application, and at that same moment, of course, the teeth and adjacent parts are offering the greatest resistance.

But is was effective in that it did open a slight space, and that it did no injury to the soft tissues.

About five or six years ago Dr. J. Lowe Young introduced to orthodontists a silk which he had obtained from France, which, unlike any other silk, would shrink when brought in contact with moisture. This at once introduced a valuable ligature, which, unlike the wire ligature which will loosen as a tooth moves, will keep tight by contracting.

It was not strange that men who adopted this silk ligature in orthodontia should essay to separate teeth with it, as they had been doing with the wire ligature, by tying it around the molar contact points.

The writer, in attempting to do this, soon discovered two things: first, that whereas it is often difficult to force even a wire ligature under the contact points, it is almost never possible to do so with the silk, as it would not be stiff enough. Again, it could not be forced down between and past the contact points, because it is too stiff or too hard. In this dilemma he thought of an old trick that he had used for a totally different purpose, and by looping the *hard* silk into a loop of dental floss, which is *soft*, the softer silk could be easily forced down between the contact points, after which the looped end of the harder silk could be drawn through (see illustration in January issue.) With the silk in this position, it was seen at once that instead of drawing one strand of the silk through, and then tying it around the contact points single, as had been the custom with the wire ligature, it would be easier, and doubly effective to utilize the loop, pass one end through, draw it tight, and tie. By this means we have two thicknesses of the ligature, and the loops of the silk under the contact point, contracting as they do in opposite directions, greatly enhances the force exerted.

This method of separating, used first for opening spaces for molar bands, was subsequently tried in operative procedures, with such success that it was communicated to friends. Among orthodontists it was found that other men had arrived at the same method, by exactly similar processes of reason applied to every-day work. Hence, it is impossible to say what man actually did it first, nor is that a matter of any great consequence.

What is of importance is the undoubted fact that it is the most effective and most painless method of separating teeth yet introduced. The silk is to be obtained in various sizes, and is effectual for separating teeth in sizes Nos. 3, 4 and 5. No. 5 silk may be used on both sides of a sixth year molar, and on both sides of the jaw (or on both jaws, on one side)—thus introducing four separations at the same time, in the mouths of sensitive children with protests rare, while the space obtained will always be adequate. The No. 5 silk is rather coarse for incisors, but the finer silks operate similarly. Where cavities are present, they may be first filled with gutta percha, or Dr. Dunn's method of placing absorbent cotton within the tie of the ligature may be adopted.

But again we say this method, which involves use of this particular silk tied in the manner described and illustrated in the January issue, is the best and most painless method of separating teeth yet evolved. It is effective because results may be obtained in twenty-four hours or less. It is painless because it obeys the following axiom:

*If we introduce the least stress against the greatest resistance in such a manner as to produce a slowly increasing stress against a slowly decreasing resistance, we attain our end with the minimum of pain.*

This ligature tied as described exerts a slight stress only at first, but this stress slowly increases, and as the teeth slowly move apart we have a slowly decreasing resistance. Moreover, there is no pressure against the gingiva.—*Items of Interest.*

